# Preparation and Characterization of Modified Polysulfone with Crosslinked Chitosan–Glutaraldehyde MWCNT Nanofiltration Membranes, and Evaluation of Their Capability for Salt Rejection

**DOI:** 10.3390/polym14245463

**Published:** 2022-12-13

**Authors:** Ahmed A. Alshahrani, Abeer A. El-Habeeb, Noor H. Alotaibi, Anfal A. Shaman, Wajd F. Almutairi, Samar M. Alotaibi, Hassan M. Hassan, Ibrahim Hotan Alsohaimi

**Affiliations:** 1 Nuclear Science Research Institute, King Abdul Aziz City for Science and Technology, P.O. Box 6086, Riyadh 11442, Saudi Arabia; 2 Department of Chemistry, College of Science, Princess Nourah bint Abdulrahman University, P.O. Box 84428, Riyadh 11671, Saudi Arabia; 3 Chemistry Department, College of Science, Jouf University, P.O. Box 2014, Sakaka 72341, Saudi Arabia

**Keywords:** nanofiltration, chitosan, glutaraldehyde, carbon nanotubes, salt rejection

## Abstract

Nanofiltration membranes were successfully created using multi-walled carbon nanotubes (MWCNTs) and MWCNTs modified with amine (MWCNT-NH_2_) and carboxylic groups (MWCNT-COOH). Chitosan (CHIT) and chitosan–glutaraldehyde (CHIT-G) were utilized as dispersants. Sonication, SEM, and contact angle were used to characterize the as-prepared membranes. The results revealed that the type of multi-walled carbon nanotubes (MWCNT, MWCNT-COOH and MWCNT-NH_2_) used as the top layer had a significant impact on membrane characteristics. The lowest contact angle was 38.6 ± 8.5 for the chitosan-G/MWCNT-COOH membrane. The surface morphology of membranes changed when carbon with carboxylic or amine groups was introduced. In addition, water permeability was greater for CHIT-G/MWCNT-COOH and CHIT-G/MWCNT-NH_2_ membranes. The CHIT-G/MWCNT-COOH membrane had the highest water permeability (5.64 ± 0.27 L m^−2^ h^−1^ bar^−1^). The findings also revealed that for all membranes, the rejection of inorganic salts was in the order R_(NaCl)_ > R_(MgSO4)_.

## 1. Introduction

Water desalination is a critical component in the fight against water constraints. There are approximately 16,880 desalination facilities worldwide [1,2,3]. Membrane technologies offer a cost-effective and ecologically beneficial way of water desalination [4]. Particle size exclusion is used in conventional filter membranes, microfiltration, and ultrafiltration, which separate pollutants by particle size. For ion and salt separation, however, nanofiltration membranes and reverse osmosis are often used [5].

Flexible polymeric membranes are well known. As a result, polymeric membranes for various water purification applications and conditions may be simply manufactured. Additionally, polymeric membranes have adjustable pore widths in contrast to ceramic membranes [6]. As a result, polymeric membranes could be used in a variety of industrial treatment methods to remove minor ions and microelements from water. For water pollution filtering, many polymeric membrane forms can be utilized. These comprise nanofiltration NF membranes used in the filtration of contaminated water, brackish water and desalination [6]. Because of their simplicity of usage, polymeric membranes are the most extensively utilized substances in membrane production. As a result, conventional methods of membrane synthesis have been developed and executed to achieve greater permeability of water and salt rejection [7]. However, polymeric membranes have chemical resistivity and lower temperature ranges than ceramic membranes, which reduce their operating lifetimes [8,9]. To increase their applicability under harsh conditions, polymeric membranes with strong thermal and chemical durability must be produced [9]. As a result, there is a need to produce membranes entirely from biopolymers that may decompose totally after use. Water filtration has employed natural biodegradable polymers such as cellulose, chitosan, starch, pullulan, glycerin, and glycoprotein [10].

Non-biodegradable polymeric membranes have been developed utilizing a broad range of polymers. Significantly, almost all industrial membranes are synthesized and manufactured with non-biodegradable substances such as polysulfone (PSf) polyvinylidene fluoride (PVDF) [11,12,13], polyethersulfone (PES) [14], polypropylene [15], polyvinyl alcohol, and polystyrene. The majority of the polymeric materials utilized in the manufacture of non-biodegradable membranes have a hydrophobic nature. Unfortunately, these membranes are vulnerable to membrane fouling since they are hydrophobic [11,12,14].

As a result, membrane quality and longevity are dramatically diminished [16,17]. Biopolymers, on the other hand, are hydrophilic, durable, recyclable, low cost, biodegradable, compostable, environmentally friendly, and non-toxic with excellent biocompatibility [18,19]. Additionally, hydrophilic substances significantly improve the rejection and efficiency of biopolymeric membranes, and biopolymers are thought to be hydrophilic [20,21]. Chitosan is one such compound that has the ability to considerably improve mechanical characteristics [22]. It is noteworthy owing to its unique chemical and biological features [23], as well as its high reactivity due to the abundance of –NH_2_ and –OH moieties [23,24]. Furthermore, chitosan has an excellent membrane-forming ability, producing membranes that have a high-water permeability. Due to the active amino and hydroxyl moieties in chitosan films, they are easily modified [25]. However, biopolymeric membranes are structurally non-biodegradable versions, requiring combining, crosslinking, and the addition of nanofillers [26]. 

In addition to their minimal biofouling characteristics, MWCNTs have shown considerable separation efficacy with inner diameters as small as 0.4 nm [27]. As a result, CNT membranes show tremendous promise in the area of water decontamination, particularly in the desalination process. Dumée et al. [28] produced a self-supporting membrane with 99 percent salt rejection and a mass flow of 12 kg/m^2^h. Additionally, CNTs can be distributed in suitable solvents using a sonicator to create composite membranes with high transport rates and large-scale nanotube applications. Using carbon nanotubes, several experiments were conducted to improve the mass transfer rate of polymer membranes [27,29,30,31,32,33]. In addition, to avoid the aggregation of CNT particles and enhance the interaction between the graphitic surface of the nanomaterial wall and the polymeric matrix, carbon nanotubes were functionalized. Crosslinking could be both non-covalent or covalent [28]. In this study, nanofiltration membranes were proposed and evaluated for salt rejection. To produce and test nanofiltration membranes functionalized with MWCNT, MWCNT-NH_2_, and MWCNT-COOH, a variety of physicochemical techniques were applied, including contact angle, SEM, and crossflow filter evaluation. These nanofiltration membranes were investigated for their capacity to remove salt from water.

## 2. Experimental

### 2.1. Materials

The PS support (PS-20) layer was purchased from Sepro Membranes Inc., Oceanside, CA, USA. The medium-molecular-weight chitosan (CHIT), glutaraldehyde (G), MgSO_4_ and NaCl were purchased from Sigma-Aldrich Co, St. Louis, MO, USA. Grafen Chemical Industries (Ankara, Turkey) provided MWCNTs (KNT-M31), or multi-walled carbon nanotubes. Carbon nanotubes with COOH functionalization (MWCNTs-COOH) were bought from Nanolab Incorporation, Waltham, MA, USA. We purchased glacial CH_3_COOH, and hydrochloric acid (HCl, 32%) from Scharlau Chemie, Barcelona, Spain. Sodium hydroxide (NaOH) was bought from Merck, Darmstadt, Germany. All of the reagents used in this investigation were made utilizing Milli-Q^®^ water, which has a resistivity of 18.2 MΩ cm.

### 2.2. Synthesis of Crosslinked Chitosan Solution

In 500 mL of an aqueous solution comprising 1% (*v*/*v*) CH_3_COOH, 0.5 g of CHIT (0.5% *w*/*v*) was dissolved to create the CHIT solutions. The mixtures were heated at 80 °C for three hours and agitated for 24 h to thoroughly solubilize the chitosan. The solutions were then allowed to cool at 21 °C overnight. To remove any non-soluble CHIT particles, the homogeneous blends were filtered over a 5.0 μm hydrophobic PTFE membrane. By adding 0.025g (0.1% *w*/*v*) of glutaraldehyde to the chitosan solution (15 mL), crosslinked chitosan was created.

### 2.3. Synthesis of Dispersions

To produce CHIT-G/MWCNT dispersion, MWCNTs (15 mg) were introduced to the CHIT-G (15 mL) solution and sonicated for 20 min. Similar to this, 15 mg each of MWCNT-COOH and MWCNT-NH_2_ was dispersed in 15 mL of CHIT-G solution to produce the CHIT-G/MWCNT-COOH and CHIT-G/MWCNT-NH_2_ dispersions.

### 2.4. Fabrication of Membranes

Using a Gardco Automatic Drawdown Machine II (DP-8301, Temecula, CA, USA), a CHIT solution (10 mL) was applied to the polysulfone (PSf) sheet (40 cm^2^) to create a top layer of thin film. To regulate membrane thickness, the Microm II Film Applicator slot height was set to 0.1 mm. The nanocomposite membrane was left to air dry. The CHIT/PS membrane was then soaked for 30 min in sodium hydroxide solution (1%). The membrane was kept in DI water until examination after being rinsed with DI water several times. Similar membranes were created for CHIT-G, CHIT-G/MWCNT, CHIT-G/MWCNT-COOH, and CHIT-G/MWCNT-NH_2_.

### 2.5. Instrumentation

The assimilation of all suspension setups (chitosan–glutaraldehyde/MWCNT, chitosan–glutaraldehyde/MWCNT-COOH and chitosan–glutaraldehyde/MWCNT-NH_2_) was investigated utilizing a Cary 500 UV–vis–NIR spectrophotometer (Richmond, VA, USA) from 300 to 1000 nm. In a tiny vial (20 mL), Milli-Q water (10 mL) was used to dilute all of the dispersion setups (50 μL). At 21 °C, the spectra were estimated using the scattering configurations in a quartz cuvette with a 1 cm channel length. FTIR spectroscopy and elemental analysis were employed to verify the chemical structures of ASMR and the prepared intermediates.

A Schottky field outflow checking electron magnifying lens (SFESEM) (JSM—7610F JEOL Ltd., Tokyo, Japan) was used to inspect the surface morphologies and cross areas of the layers. Utilizing a JEOL JEC—3000FC fine coating technique (JEOL Ltd., Tokyo, Japan), each layer was coated twice for 30 s. Each layer was put on a stub containing carbon particles. The prepared materials were tested at various amplifications utilizing speed increase voltages varying from 5 to 15 kV. C. Using Milli-Q water, the contact-angle technique was used to measure membrane wettability and hydrophilicity. A smooth membrane surface (4 × 35 mm) was held flat on a glass slide. Using a microsyringe, a 2 µL water droplet was deposited on the membrane surface. The droplet images were taken using a U3 Series digital camera. The Ramé-hart DROPimage Advanced programmer was then used to calculate the contact angle between the droplet and membrane surface. On a few randomly chosen regions on the surface of each membrane, this technique was used to guarantee the measurement’s accuracy and error rate.

### 2.6. Membrane Performance

The efficiency of the prepared membranes was examined utilizing a crossflow filtration system (Sterlitech^TM^ CF042 Membrane Test Skid, Sterlitech Corp., Auburn, WA, USA). The exact membrane area is 42 cm^2^. The rate of flow of the feed was tuned at ≈ 7 L/min. The feed water had a salt content of 2000 ppm (NaCl and MgSO_4_). After 30 min of water filtration studies, all findings for the water permeability and salt rejection were collected in order to produce a steady-state operation. The membrane pressure readings of 6, 8, 10, 12, 14, 18, and 22 bar were utilized to report the findings of the rejection experiments, which were conducted at pH 7. All experiments on the permeability and rejection characteristics of manufactured membranes were conducted at 20 °C.

The water flux (*J*) was estimated utilizing Equation (1) [30,31]:

(1) $$J=\left(\frac{1}{A}\right)\frac{dv_{p}}{dt}$$

where $v_{p}$, *A*, and *t* are, respectively, volume of permeate (L), effective membrane area (m^2^), and time (h).

Salt rejection, *R*, is determined using Equation (2) [30,31]:

(2) $$R_{o}=\left(1-\frac{C_{p}}{C_{f}}\right)*100\;$$

where $C_{p}$ and $C_{f}$ are the salt concentrations in the permeate and feed streams, respectively. 

## 3. Results and Discussion 

### 3.1. Synthesis of MWCNTs, MWCNTs-COOH and MWCNTs-NH_2_ Dispersions Containing Crosslinked Chitosan 

**Figure 1:**
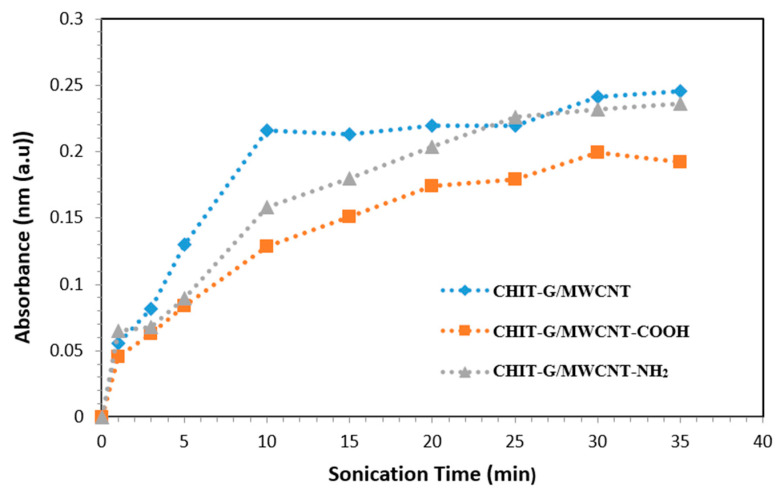
Effects of longer sonication times on the absorbance of MWCNT dispersions at 660 nm.

The production of dispersions including MWCNTs, MWCNTs-COOH, and MWCNTs-NH_2_ with CHIT-G was monitored using absorption spectrophotometry. We were able to create MWCNT dispersions utilizing ultrasonic for only a few minutes (20 min). The sonication time was a major concern as it had to be long enough to properly suspend the MWCNTs, but not so long as to cause defects in the nanotubes, shorten their length or otherwise negatively affect their properties [34,35,36]. Absorption spectrophotometry is particularly appropriate for assessing the impact of sonication time or sample conditions on the extent of MWCNT suspension. This is attributable to the fact that it is a valuable method for determining how much de-bundling of nanotubes in suspensions has occurred. The absorbance of all dispersions was measured at wavelengths ranging from 300 to 1000 nm. Increased sonication time increased absorbance that is compatible with other investigations in the literature [37,38]. To find an acceptable sonication duration for forming the other forms of suspensions, the absorbance at a single wavelength (660 nm) was evaluated as a function of sonication time as shown in Figure 1. To prevent absorbance due to the solvent and the dispersant (chitosan or crosslinked chitosan), a wavelength of 660 nm was chosen. The absorbance at 660 nm changed for each suspension in response to increasing sonication duration, as shown in Figure 1. After 10, 20, and 25 min of sonication for the CHIT-G/MWCNTs, CHIT-G/MWCNT-COOH and CHIT-G/MWCNT-NH_2_, respectively, absorbance had reached or was approaching a plateau zone. This suggests that the time frame was long enough to create a well-dispersed MWCNT sample suitable for PS membrane preparation. When the sonication period was extended, the absorbance at 660 nm did not change much again.

### 3.2. Morphology of Membrane

**Figure 2:**
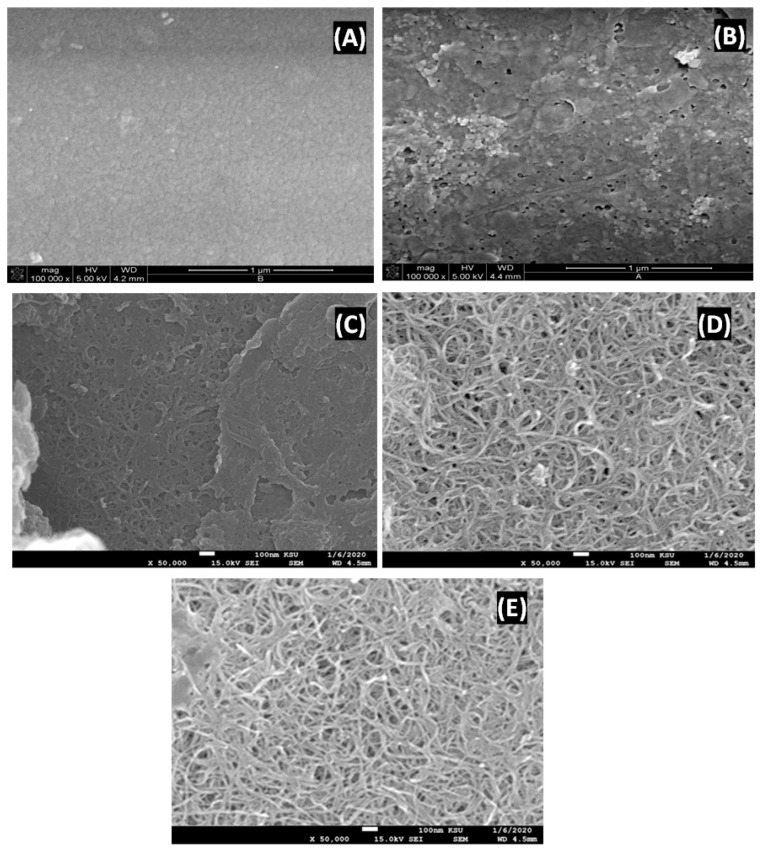
SEM images of the five PSf membranes: chitosan (**A**), CHIT- G (**B**), CHIT-G/MWCNT (**C**); CHIT-G/MWCNT-COOH (**D**) and CHIT-G/MWCNT-NH_2_ (**E**).

Figure 2 displays SEM images of the membranes synthesized in this investigation. In the instance of the PSf membrane that has been coated with chitosan (Figure 2A), nanopore creation takes place during the coating process. When chitosan is crosslinked in the existence of glutaraldehyde, it often results in phase separation, which leads to pore development [39] (Figure 2B). In comparison to the MWCNT/CHIT-G/PS membranes (Figure 2C), SEM images of MWCNTs-COOH/CHIT/PS (Figure 2D) and MWCNTs-NH_2_/CHIT/PS (Figure 2E) membranes displayed a multitude of nanotubes that were tightly intertwined on their surface. This is due to the CHIT chain wrapping around MWCNTs-COOH and MWCNTs-NH_2_ as a consequence of the COOH and NH_2_ groups on MWCNT walls interacting with the CHIT-NH_2_ chain and -OH moieties of the CHIT chain [40]. CHIT’s quiet compatibility with MWCNTs-COOH and MWCNTs-NH_2_ is consistent with prior studies [40]. On the other hand, we can assume that the content of MWCNTs in chitosan is smaller than the content of MWCNTs-COOH, which is mainly owing to the limited molecular attraction between MWCNTs and CHIT along with the hydrophobic character of MWCNTs [40,41]. As a result, MWCNTs in the CHIT matrix have a propensity to group together. MWCNTs are thus less equally distributed in the CHIT matrix than MWCNTs-COOH and MWCNTs-NH_2_.

### 3.3. Contact Angle

The surface nature of the membrane has a significant influence on its performance [42,43]. The hydrophilicity of the surface is an essential property that determines membrane efficiency [42,43,44]. It profoundly influences how aqueous, organic, and inorganic colloidal particles interact with the membrane surface [45,46]. The static contact-angle experiment is used to assess the hydrophobicity and hydrophilicity of surfaces [47]. When the surface contact angle decreases, it typically suggests that the surface’s hydrophilicity has improved. 

The contact angles of all PSf membranes are presented in Table 1. From these results, we found that:

- The un-crosslinked chitosan/PSf membrane has a remarkably greater contact angle (72.8 ± 4.9°) than all the other PSf membranes which contained crosslinked chitosan.
- The contact angles of the CHIT-G/MWCNT/PSf, CHIT-G/MWCNT-NH_2_/PSf and CHIT-G/MWCNT-COOH/PSf membranes were recorded as 42.4°, 40.0°, and 38.4°, respectively. These values were lower than that for the CHIT-G/PS membrane. This suggests that the dispersion of MWCNTs in the CHIT matrix enhanced its hydrophilicity [44]. Due to the existence of carboxylic and amino moieties on the surfaces of MWCNTs, these results also demonstrate that the CHIT-G/MWCNT-NH_2_/PSf and CHIT-G/MWCNT-COOH/PSf membranes are more hydrophilic than the CHIT-G/MWCNT/PSf membrane. As a result, this enhances MWCNT dispersion in the polymers and aqueous solution, as well as membrane hydrophilicity [42].
- The contact angles obtained in the present research are smaller to those previously published for membranes synthesized by utilizing MWCNTs [30,32]. This may be due to the use of MWCNT-COOH and MWCNT-NH_2_ and glutaraldehyde (G) for crosslinking chitosan.

### 3.4. Membrane Performance

#### 3.4.1. Permeability

**Figure 3:**
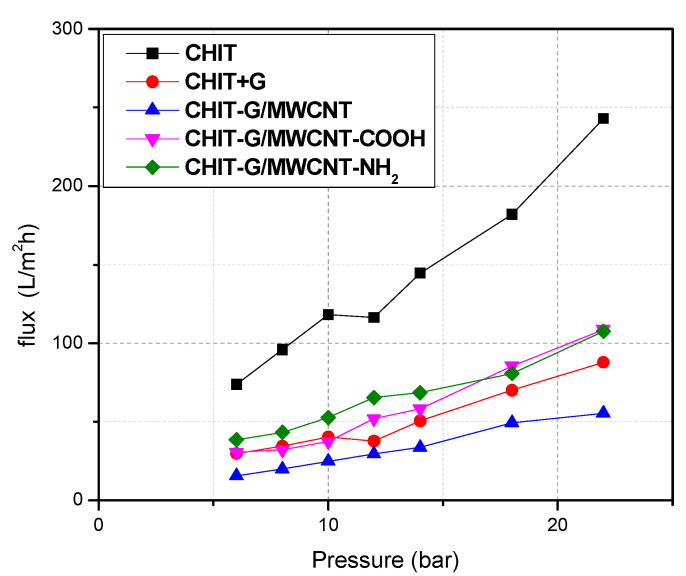
Flux plotted against transmembrane pressure for membranes.

Membrane hydrophilicity, chemical composition, and surface charge all have a major influence on the water flow rate because they change the combination between the solution and membrane surface [48]. Figure 3 shows the dependence of the flux on the pressure on the membrane. The permeability of all membranes was determined utilizing the trend line of working pressure and permeate flow. With greater working pressure, the fluxes of the permeate of all PSf membranes increased; findings are illustrated in Table 1. After each PSf membrane was used for 8 h, the CHIT-G/MWCNT-COOH/PSf membrane had higher water permeability (5.6 ± 0.3 L m^−2^ h^−1^ bar^−1^) and remarkably greater flux (Figure 3) in comparison with other membranes. This is owing to the carboxylic group’s ability to attach water molecules through hydrogen bonding. When compared to prior experiments, all of the studied membranes showed improvements in water permeability [30,32]. The water permeability of all PSf membranes prepared in this work was in the range 3.6–5.6 L m^−2^ h^−1^ bar^−1^. These findings outperform prior studies employing unsubstituted MWCNTs and other crosslinking agents.

#### 3.4.2. Salt-Rejection Capability

**Figure 4:**
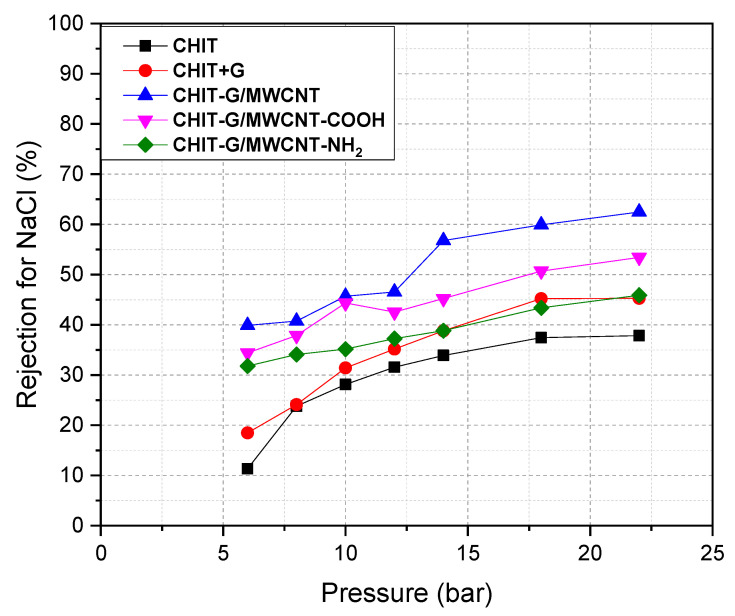
NaCl rejection at pH = 7 with membrane pressure.

**Figure 5:**
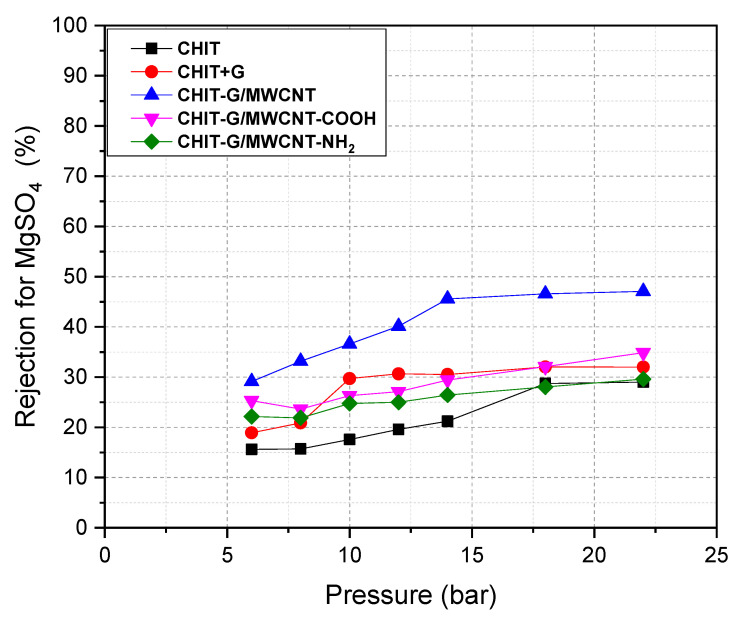
MgSO_4_ rejection at pH = 7 with membrane pressure.

Salt rejection by all PSf membranes was examined utilizing a crossflow RO/NF system with NaCl and MgSO_4_ solutions (as a single salt solution (2000 ppm), and tests were conducted at 20 °C with varied working pressure. Salt rejection by all PSf membranes (as indicated in Equation (2) in the Experimental Part) was drawn versus permeate flow (Figure 4 and Figure 5). In general, as the operating pressure increased, salt rejection increased. More significantly, Figure 4 and Figure 5 demonstrate an exponential relation between working pressure and salt rejection. The NaCl and MgSO_4_ solutions exhibit a dramatic rise in salt rejection with increasing operating pressure. The rejection of NaCl using CHIT-G/MWCNT/PSf, CHIT-G/MWCNT-NH_2_/PSf and CHIT-G/MWCNT-COOH/PSf membranes was highest (38–62%) compared to other membranes (CHIT/PSf and CHIT-G/PSf). Additionally, these membranes displayed higher MgSO_4_ rejection (Roughly 29–49%) than the CHIT/PSf (15–29%) and CHIT-G/PSf (19–30%) membranes. It should be noted that MgSO_4_ rejection by PSf membranes was quite lower than by NaCl. The order of rejection reported here coincides with the hydration ionic radius of Na^+^ (0.360 nm) < Mg^2+^ (0.395 nm). There is no information in the literature on the ionic radius of SO_4_^2-^. Our findings agree well with a previous investigation by Tongwen et al. [49], who also reported that inorganic salt rejection was in the order R_(NaCl)_ > R_(MgSO4)_. The rejection of NaCl and MgSO_4_ by the CHIT-G/MWCNT/PSf membrane was also somewhat higher than that of the CHIT-G/MWCNT-NH_2_/PSf and CHIT-G/MWCNT-COOH/PSf membranes, as shown in Figure 4 and Figure 5. This could be related to the porosity and columbic repulsion for unsubstituted MWCNTs, which may lead to increased rejection. These findings are in line with the SEM images of the prepared PSf membranes.

## 4. Conclusions

Multi-walled carbon nanotubes (MWCNTs) were used with chitosan and crosslinked chitosan to create PSf membranes for applications requiring nanofiltration. We performed a comparison of the properties of different membranes. A technique for enhancing MWCNT dispersion in water was offered by a crosslinked chitosan (CHIT-G) dispersant. The dispersion of MWCNTs in (CHIT-G) increased the hydrophilicity and stability of the membrane surface, according to contact angle experiments for PS membranes. Under identical operating conditions, the salt rejection of three PS membranes was in the order R_(NaCl)_ > R_(MgSO4)_. These findings suggest that the unhydrated radius of these inorganic ions is primarily responsible for controlling the separation of these ions. On the other hand, the carboxylic group has a significant influence on the properties and functionality of the CHIT-G/MWCNTs-COOH/PSf membrane. Due to the link between the -COOH group on the MWCNT walls and the functional moieties on the CHIT matrix, MWCNTs-COOH are better diffused in CHIT-G than the un-functionalized MWCNTs. In addition, compared to CHIT-G/MWCNTs/PSf and CHIT-G/MWCNTs-NH_2_/PSf membranes, the effect of the -COOH group on MWCNTs-COOH increased the hydrophilicity of the membrane. This enhances the capacity of CHIT-G/MWCNTs-COOH/PSf membranes to reject metal ions while maintaining water permeability. This is because carboxylic (-COOH) groups have a stronger propensity to chelate metal ions, increasing the total capacity of membrane for adsorption. In summary, this research offers a workable procedure for creating membranes that are effective and environmentally friendly for the elimination of metal ions from aquatic environments.

## Figures and Tables

**Table 1:** Contact angles and water permeability.

PS Membrane	Contact Angle (°)	Permeability (L m^−2^ h^−1^ bar ^−1^)
CHIT	72.8 ± 4.9	10.03 ± 0.8
CHIT-G	57.4 ± 5.1	3.7 ± 0.4
CHIT-G/MWCNT	42.4 ± 3.4	2.6 ± 0.1
CHIT-G/MWCNT-COOH	38.6 ± 8.5	5.3 ± 0.4
CHIT-G/MWCNT NH_2_	40.0 ± 5.6	4.2 ± 0.3
MWCNT/chitosan-glycerin ^a^	80 ± 2.0	0.6 ± 0.02
MWCNT/chitosan-PEGD ^a^	76 ± 3.0	0.2 ± 0.01
CNTs-COOH/CHIT/PS ^b^	59.4 ± 5.4	3.9 ± 0.5

^a^ Data from [32]. ^b^ Data from [30].

## Data Availability

The data presented in this study are available on request from the corresponding author.
